# Pipeline for amplifying and analyzing amplicons of the V1–V3 region of the 16S rRNA gene

**DOI:** 10.1186/s13104-016-2172-6

**Published:** 2016-08-02

**Authors:** Heather K. Allen, Darrell O. Bayles, Torey Looft, Julian Trachsel, Benjamin E. Bass, David P. Alt, Shawn M. D. Bearson, Tracy Nicholson, Thomas A. Casey

**Affiliations:** 1 Food Safety and Enteric Pathogens Research Unit, National Animal Disease Center, Agricultural Research Service, Ames, IA 50010 USA; 2 Infectious Bacterial Diseases Research Unit, National Animal Disease Center, Agricultural Research Service, Ames, IA 50010 USA; 3 Interdepartmental Microbiology Graduate Program, Iowa State University, Ames, IA 50010 USA; 4 Diamond V, Cedar Rapids, IA 52404 USA; 5 Virus and Prion Research Unit, National Animal Disease Center, Agricultural Research Service, USDA, Ames, IA 50010 USA

**Keywords:** V1–V3, 16S rRNA gene, MiSeq, Mock community, Microbial ecology

## Abstract

**Background:**

Profiling of 16S rRNA gene sequences is an important tool for testing hypotheses in complex microbial communities, and analysis methods must be updated and validated as sequencing technologies advance. In host-associated bacterial communities, the V1–V3 region of the 16S rRNA gene is a valuable region to profile because it provides a useful level of taxonomic resolution; however, use of Illumina MiSeq data for experiments targeting this region needs validation.

**Results:**

Using a MiSeq machine and the version 3 (300 × 2) chemistry, we sequenced the V1–V3 region of the 16S rRNA gene within a mock community. Nineteen bacteria and one archaeon comprised the mock community, and 12 replicate amplifications of the community were performed and sequenced. Sequencing the large fragment (490 bp) that encompasses V1–V3 yielded a higher error rate (3.6 %) than has been reported when using smaller fragment sizes. This higher error rate was due to a large number of sequences that occurred only one or two times among all mock community samples. Removing sequences that occurred one time among all samples (singletons) reduced the error rate to 1.4 %. Diversity estimates of the mock community containing all sequences were inflated, whereas estimates following singleton removal more closely reflected the actual mock community membership. A higher percentage of the sequences could be taxonomically assigned after singleton and doubleton sequences were removed, and the assignments reflected the membership of the input DNA.

**Conclusions:**

Sequencing the V1–V3 region of the 16S rRNA gene on the MiSeq platform may require additional sequence curation in silico, and improved error rates and diversity estimates show that removing low-frequency sequences is reasonable. When datasets have a high number of singletons, these singletons can be removed from the analysis without losing statistical power while reducing error and improving microbiota assessment.

**Electronic supplementary material:**

The online version of this article (doi:10.1186/s13104-016-2172-6) contains supplementary material, which is available to authorized users.

## Background

The affordability and scalability of nucleic acid sequencing have enabled researchers to conduct microbial community analyses on an unprecedented scale. One highly used method to query bacterial communities involves sequencing amplicons of the 16S rRNA gene. However, sequence and analysis of these amplicons has numerous technical limitations including chimera formation during the PCR step and errors introduced by sequencing technologies. Previous advances in validating 16S rRNA gene sequence analyses have employed various sequencing platforms, multiple sequencing centers, and both real and mock bacterial communities [1, 2]. In silico methods, such as chimera removal [3, 4], quality filtering [5], and clustering methods [6], have been developed and validated to improve analysis of amplicons and are essential to separate true data from noise [7].

The quality of 16S rRNA gene sequence data is dependent on numerous technical steps that are difficult to control, often resulting in thousands of unique sequences even after implementing quality-control steps. One approach to managing these low-frequency sequences is to implement closed-reference operational taxonomic unit (OTU)-picking, which clusters sequences into OTUs based on their assignment to a reference database [8]. However, many researchers choose not to require phylogenetic assignment prior to clustering because it could eliminate important, undescribed members of the community. An additional approach is to remove low-frequency OTUs after sequence clustering, although it is computationally expensive to retain sequencing artifacts through distance matrix creation and subsequent clustering. Unreferenced removal of sequences prior to OTU-calling improves the speed and feasibility of analyzing large datasets, as we demonstrate here by sequencing and analyzing the 16S rRNA gene, V1–V3 region, of an artificial (mock) microbial community.

The challenge of sequencing 16S rRNA gene amplicons via the MiSeq platform is choosing a variable region that both informs the microbiota of interest and results in an amplicon sufficiently short to overlap both forward and reverse reads of a paired-end reaction. The V4 region has therefore been used for this sequencing method because its short amplicon (~400 bp including primer sequences) is technically amenable to assembly with a low error rate (0.01 %) [2]. However, longer 16S gene regions are sometimes preferred for biological reasons based on the research question despite the potential higher error rates associated with longer amplicon sequences. For example, the V1–V3 region, but not the V3–V5, region can discriminate *Staphylococci* populations [9]. We have previously used the V1–V3 region to analyze the swine gut microbiota (e.g., [10]), thus we adapted the MiSeq amplicon method for this region by adding a singleton removal step prior to OTU clustering and validated the method by sequencing 12 technical replicates of a mock community.

## Methods

### Generating the mock community

Members of the mock community were selected based on three criteria: (1) diversity, (2) availability of a whole genome sequence, and (3) availability of genomic DNA either in-house or from DSMZ (Leibniz Institute DSMZ-German Collection of Microorganisms and Cell Cultures https://www.dsmz.de/home.html). The following equation was used to balance the contribution of each microbial genome (19 bacteria, 1 archaeon, Table 1) to the mock community: (target #16S rRNA gene copies in stock mock)/(#16S rRNA gene copies per genome × #genome copies per microgram). Genomic copies of the 16S rRNA gene from each member comprised 5 % of the total mock community. The mock community was stored in aliquots at −20 °C until 16S rRNA gene amplification.

**Table 1:** 16S gene composition of the mock community

	Strain	Genome size^a^	# 16S genes^a^	Genome copies per microgram^b^	Reference or DSMZ catalogue number^c^
1	*Campylobacter jejuni* 11168	1,641,481	3	5.64E+08	[23]
2	*Salmonella enterica* serovar Typhimurium SL1344	4,878,012	6	1.89E+08	[24]
3	*Escherichia coli* mg1655	4,656,144	7	1.99E+08	[25]
4	*Megasphaera elsdenii* LC-1	2,474,718	7	3.74E+08	DSM 20460
5	*Cloacibacillus porcorum* CL-84	3,585,187	3	2.57E+08	[26]
6	*Brachyspira hyodysenteriae*	3,050,489	1	3.04E+08	[27]
7	*Haemophilus parasuis* 29755	2,224,137	2	4.17E+08	[28]
8	*Bordetella bronchiseptica* 1289	5,207,899	3	1.78E+08	[29]
9	*Staphylococcus aureus* USA300	2,872,915	5	3.22E+08	[30]
10	*Bacteroides thetaiotaomicron*	6,293,399	5	1.47E+08	DSM 2079
11	*Faecalibacterium prausnitzii* A2-165	3,080,849	3	3.01E+08	DSM 17677
12	*Streptococcus parasanguinis*	2,153,652	4	4.30E+08	DSM 6778
13	*Parabacteroides merdae*	4,431,877	3	2.09E+08	DSM 19495
14	*Oscillibacter valericigenes*	4,470,622	3	2.07E+08	DSM 18026
15	*Desulfovibrio gigas*	3,788,225	4	2.45E+08	DSM 1382
16	*Lactobacillus delbrueckii* subspecies bulgaricus	1,864,998	9	4.97E+08	DSM 20081
17	*Coriobacterium glomerans*	2,115,681	2	4.38E+08	DSM 20642
18	*Oxalobacter formigenes* BA-2	2,509,362	1	3.69E+08	DSM 4420
19	*Roseburia hominis* A2-183	3,592,125	4	2.58E+08	DSM 16839
20	*Methanobrevibacter smithii* ^d^	1,704,865	1	5.43E+08	DSM 2375

^a^All except *C. porcorum* were calculated by the Joint Genome Institute’s Integrated Microbial Genomes Database https://img.jgi.doe.gov/cgi-bin/w/main.cgi. *C. porcorum* was calculated manually (BioProject PRJNA335387)

^b^Estimates were calculated by URI Genomics & Sequencing Center http://cels.uri.edu/gsc/cndna.html

^c^Genomic DNAs were acquired from DSMZ, the Leibniz Institute DSMZ-German Collection of Microorganisms and Cell Cultures https://www.dsmz.de/home.html

^d^This archaeon was included as a control for non-specific amplification

### Generating and sequencing 16S rRNA gene sequence amplicons

Twelve replicates of the mock community were added to a single well of each of 12 96-well plates alongside hundreds of experimental samples for phylotype analysis. The 16S rRNA gene V1–V3 region was amplified using previously published primers [11] fused to barcodes [2] and adaptor sequences for the MiSeq instrument (Illumina Inc., San Diego, CA). Amplicon libraries were generated according to Kozich et al. [2]. Briefly, PCRs contained the following: 17 µl AccuPrime Pfx SuperMix (Life Technologies, Grand Island, NY), 5.0 µM each of the primers i5+V3 and i7+V1 (Table 2), and 25 ng of the mock community. The following PCR conditions were used: 2 min at 95 °C, 22 cycles of [20 s at 95 °C, 15 s at 55 °C, 5 min 72 °C], 72 °C for 10 min. Libraries were normalized using the SequalPrep Normalization Plate Kit (LifeTechnologies) and quantified using both Bioanalyzer (Agilent Technologies, Santa Clara, CA) and Kapa SYBR Fast qPCR (Kapa Biosystems, Wilmington, MA). Normalized pools were sequenced using version 3 (300 × 2) chemistry on the MiSeq instrument (Illumina) according to manufacturer’s instructions [2].

**Table 2:** Primers used in this study

Primer name	Sequence (5′–3′)^a^
i5+V3	**AATGATACGGCGACCACCGAGATCTACAC** ATCGTACGTATGGTAATTCA*ATTACCGCGGCTGCTGG*
i7+V1	**CAAGCAGAAGACGGCATACGAGAT** AACTCTCGAGTCAGTCAGCC*GAGTTTGATCMTGGCTCAG*
For.seq.V3	TATGGTAATTCAATTACCGCGGCTGCTGG
Rev.seq.V1	AGTCAGTCAGCCGAGTTTGATCMTGGCTCAG
Index.V1	CTGAGCCAKGATCAAACTCGGCTGACTGACT

^a^Illumina’s MiSeq adaptor sequences are in bold. Underlined portion denotes the barcode region. Barcodes used in this study are from the Schloss laboratory’s MiSeq SOP (http://www.mothur.org/wiki/MiSeq_SOP; [2]). Italics denotes the V1 or V3 16S rRNA gene primer [11]

### Data analysis

The mothur analysis package was used to assemble contigs, align sequences, trim sequences, remove chimeras (UCHIME, [3]), and remove non-bacterial sequences (mothur versions 1.33.3 and 1.34.0, http://www.mothur.org/wiki/MiSeq_SOP, [2, 12]). Following these quality-control steps, the data were rarified to 6654 sequences/sample and analyzed in three ways: (1) all sequences together, (2) with cross-sample singletons removed, or (3) with cross-sample singletons and doubletons removed. Cross-sample singletons and doubletons were defined as sequences that occurred only once (singletons) or twice (doubletons) among all samples. Taxonomy was assigned by aligning to mothur’s implementation of the SILVA database [13]. OTUs were clustered at 97 % similarity and analyzed in mothur for community metrics. Richness, evenness, and diversity were also conducted in mothur and included the use of the program Catchall [14]. Error analysis was performed in mothur based on the alignment of the experimental mock community to a FASTA file containing the actual 16S rRNA gene sequences from the genome sequences (Additional file 1). A complete list of the commands executed in mothur is available (Additional file 2).

## Results and discussion

### Sequencing the V1–V3 region yields spurious sequences

**Fig. 1:**
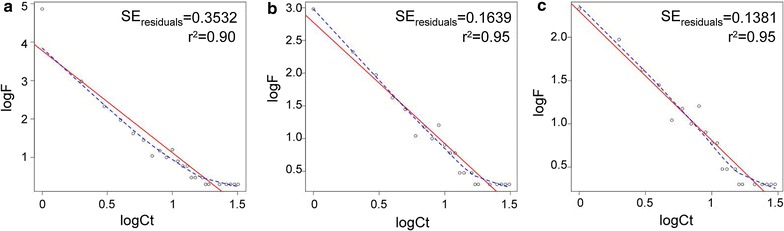
Cluster size versus frequency plot. The log_10_ of the cluster size (logCt) (i.e. number of sequences in each cluster) plotted against the log_10_ of the frequency of cluster membership sizes (i.e. the frequency of clusters that contained *n* sequences, where *n* is the cluster membership size) found among all mock community samples. Plots of all mock community data (**a**), data with cross-sample singletons removed (**b**), and data with both cross-sample singletons and doubletons removed (**c**) are shown. *Red line* regression line, *blue line* lowess fit line, *SE* standard error, r^2^ coefficient of determination

The V1–V3 region of the 16S rRNA gene was sequenced from twelve technical replicates of a mock community on the MiSeq platform. The program mothur was used to make quality-checked contigs from paired sequences, align the sequences to the SILVA database, trim the sequences, and remove chimeras. Intermediate data files from processing all 12 replicates together were large, and in total contained a high percentage (35.5 %) of sequences that failed to cluster with other sequences and were unique to an individual sampling of the mock community. The relationship between cluster membership size and the frequency of cluster sizes was explored via log/log plots to stabilize the large variability (Fig. 1a). Both linear regression and lowess fitting of the data indicated that low-frequency sequences deviate from the overall trend. We aligned the sequences to the SILVA reference database and found that the sequence clusters with one or two members were either not classifiable or assigned to bacteria not present in the mock community, and therefore were either non-informative or spurious. We removed the single sequences that did not cluster or that clustered with only one other sequence, and then plotted and analyzed the data by linear regression and lowess (Fig. 1b, c). Removal of the non-informative sequences improved the overall fit of the data.

### Removal of low-frequency sequences improves microbiota assessments

We examined the accuracy of diversity indices with and without the removal of low-frequency sequences. Removal of singletons, or singletons and doubletons, decreased the error rate and improved the accuracy of diversity metrics (Table 3). This is demonstrated by the reduction of observed and estimated number of species closer to the actual composition of the mock community. Interestingly, the two copies of the 16S rRNA gene in *Haemophilus parasuis* strain 29755 are only 94 % identical, so the 19 bacterial genomes in the mock community result in 20 bacterial OTUs.

**Table 3:** Average diversity estimates of the mock community (n = 12, rarified to 6654 sequences per sample) with and without removing low-frequency sequences

Mock community	Actual number of OTUs^a^	Observed number of OTUs	Estimated total number of OTUs^b^	Chao diversity index	Shannon diversity index	Inverse Simpson index	Error rate (%)	File size (Gb)^c^
All sequences	20	734 ± 56	374,770 ± 214,807	21,676 ± 3273	3.6 ± 0.1	18 ± 0.8	3.6	41
Singletons removed	20	28 ± 0.8	68 ± 13	41 ± 3	2.7 ± 0.02	12 ± 0.3	1.4	21
Single and doubletons removed	20	22 ± 0.3	22 ± 0.3	23 ± 0.7	2.6 ± 0.02	12 ± 0.3	1.3	3

Average diversity estimates: plus or minus (±) the standard error of the mean, where appropriate

^a^ *Haemophilus parasuis* has two divergent copies of the 16S rRNA gene that cluster separately

^b^The estimated total number of OTUs is the number of OTUs predicted to be in the sample based on the number of OTUs observed in the sequences. The program Catchall was used to make the estimates [14]

^c^Size of the distance matrix file

Removing the large number of clusters with a membership size of one sequence (or one and two sequences) has a distinct advantage for streamlining the clustering of sequences into OTUs by reducing the size of the dataset without detracting from the ability to make valid comparisons. Reducing the number of sequences resulted in a 13-fold decrease in file size and exponentially reduced the time and memory space needed to create the distance matrix since the distance matrix is comprised of pairwise comparisons (Table 3).

**Fig. 2:**
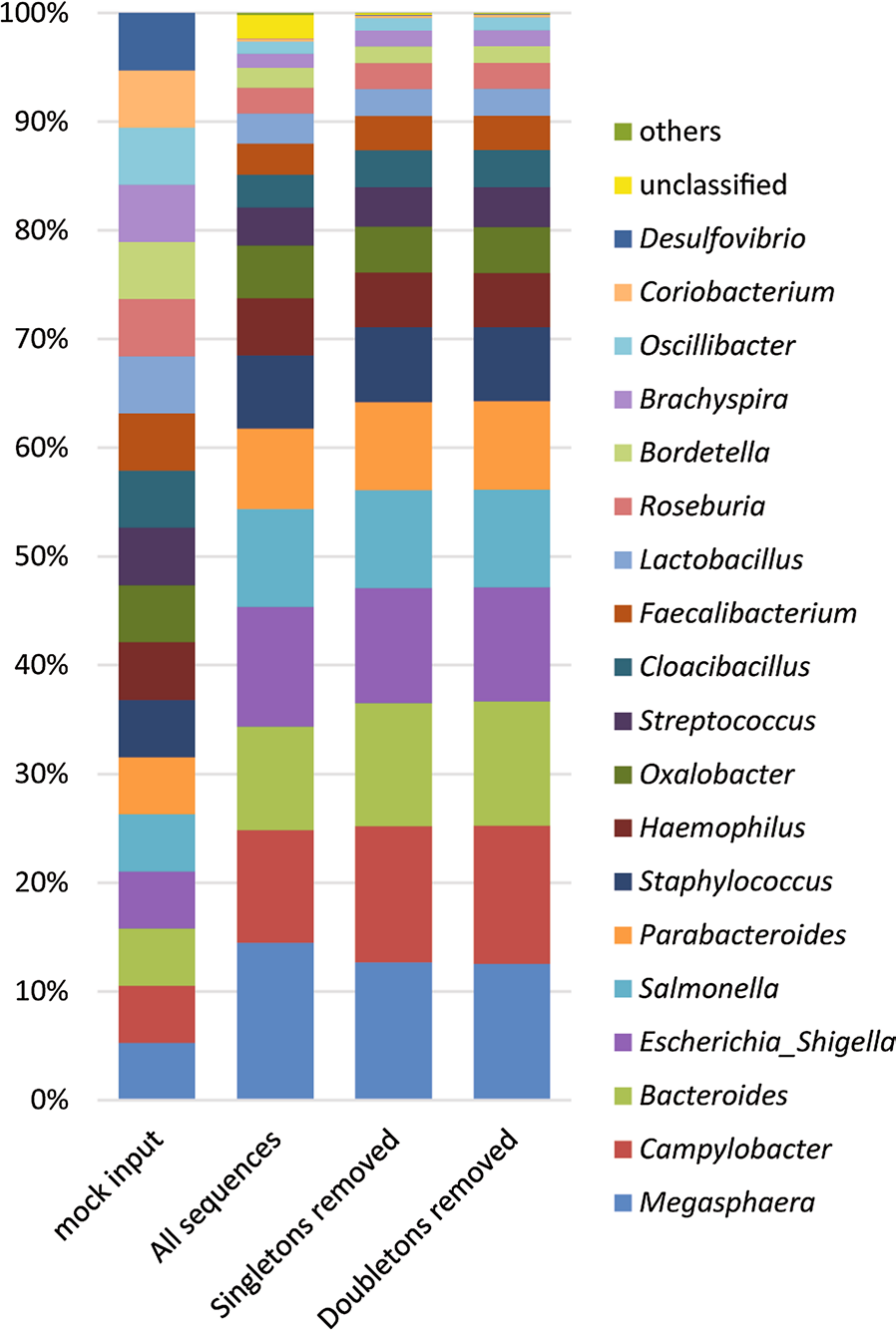
Average relative abundance of bacterial genera in 12 replicates of the mock community

OTUs were classified to assess how well the empirical taxonomy matched the known taxonomy of the input community. All members of the mock community were detected in the output (Fig. 2). *Desulfovibrio* and *Coriobacterium* were detected at only 0.05 and 0.23 % relative abundance, respectively, and subsequent analysis of their 16S rRNA gene sequences confirmed mismatches in the binding site for the V1 primer that would cause decreased amplification. This was not surprising since others have shown that PCR, not sequencing, is the largest source of bias in microbiota analyses [15, 16]. Also, thousands of sequences could not be classified despite their derivation from a defined community, perhaps because sequences from the MiSeq instrument have been shown to contain a small amount of cross-contamination between sequencing runs [17]. Removal of singletons or singletons and doubletons reduced the amount of unclassified sequences, decreasing their abundance from 2 % of the all reads to 0.1 % of the no-singletons dataset (Fig. 2). Our work shows that removing the large number of sequences that did not cluster and were unique to a single sample improved the taxonomic assignment of the sequences while preserving the relative abundance of real community members and facilitating the data analysis.

## Conclusions

Discarding low-frequency sequences can be concerning for those interested in the rare members of a bacterial community. Indeed, defining the rare microbiota in the context of sequencing errors has been explored [18–20]. Other work has supported the value of singleton removal [21, 22]. Our method is beneficial to researchers that use the Illumina MiSeq to sequence longer amplicons, such as the V1-V3 region of the 16S rRNA gene, from hundreds of samples. Removing the singletons and doubletons will suit the vast majority of projects seeking to analyze the abundant (>1 %) community organisms to draw biological conclusions.

## Additional files

10.1186/s13104-016-2172-6 FASTA file of 16S rRNA genes contained in the mock community.
10.1186/s13104-016-2172-6 Commands executed in mothur.
